# Molecular Mechanisms and Therapeutic Advances in KRAS G12C‐Mutant Non‐Small Cell Lung Cancer: Toward Mechanism‐Guided Longitudinal Management

**DOI:** 10.1002/cai2.70082

**Published:** 2026-10-15

**Authors:** Haiying Wang, Hanxiao Chen, Sheng Li, Jun Zhao

**Affiliations:** ^1^ Department of Thoracic Medical Oncology Peking University Cancer Hospital Beijing China

**Keywords:** clinical practice in China, combination therapy, drug resistance, KRAS G12C, non‐small cell lung cancer, targeted therapy

## Abstract

KRAS G12C‐mutant non‐small cell lung cancer (NSCLC) has become a clinically actionable molecular subtype. Discovery of the switch‐II pocket enabled the development of covalent KRAS G12C(OFF)‐state inhibitors, transforming a historically “undruggable” target into a therapeutic vulnerability. Drugging KRAS, however, proved to be a problem of chemistry, whereas sustaining its inhibition has proved to be a problem of biology. KRAS G12C‐mutant NSCLC is biologically heterogeneous, and the magnitude and durability of clinical benefit are shaped by co‐mutations, the tumor microenvironment, brain metastases status, pharmacologic differences between agents, and the dynamic evolution of resistance. Randomized trials have demonstrated that sotorasib and adagrasib improve progression‐free survival (PFS) and objective response rates (ORR) over docetaxel in previously treated disease, yet these gains have not translated consistently into a definitive overall survival (OS) advantage. This review synthesizes the epidemiology, molecular biology, diagnostic strategies, first‐ and later‐line treatment evidence, resistance mechanisms, and emerging KRAS/RAS‐directed approaches. We distinguish randomized or guideline‐supported practice from early‐phase and translational signals, and propose an implementable framework that combines PD‐L1, tumor mutational burden, STK11/KEAP1/TP53 status, TTF‐1 expression, brain metastases, and circulating tumor DNA (ctDNA) for risk stratification and mechanism‐guided management. At disease progression, oligoprogression should be distinguished from systemic progression, and tissue re‐biopsy together with plasma profiling should be considered when feasible. In China, several domestically developed KRAS G12C inhibitors have been approved, and reimbursement coverage is expanding; the cost of molecular testing, uneven institutional access, regional disparities and limited confirmatory and real‐world evidence nonetheless continue to constrain implementation. Future priorities include randomized first‐line comparisons, biomarker‐defined combination strategies, improved central nervous system activity and mechanism‐matched treatment after resistance.

## Graphical abstract

This review summarizes the molecular mechanisms, clinical development, resistance patterns, and emerging treatment strategies of KRAS G12C‐mutated non‐small cell lung cancer, with emphasis on approved inhibitors, combination approaches, next‐generation agents, and future directions from both global and Chinese perspectives.

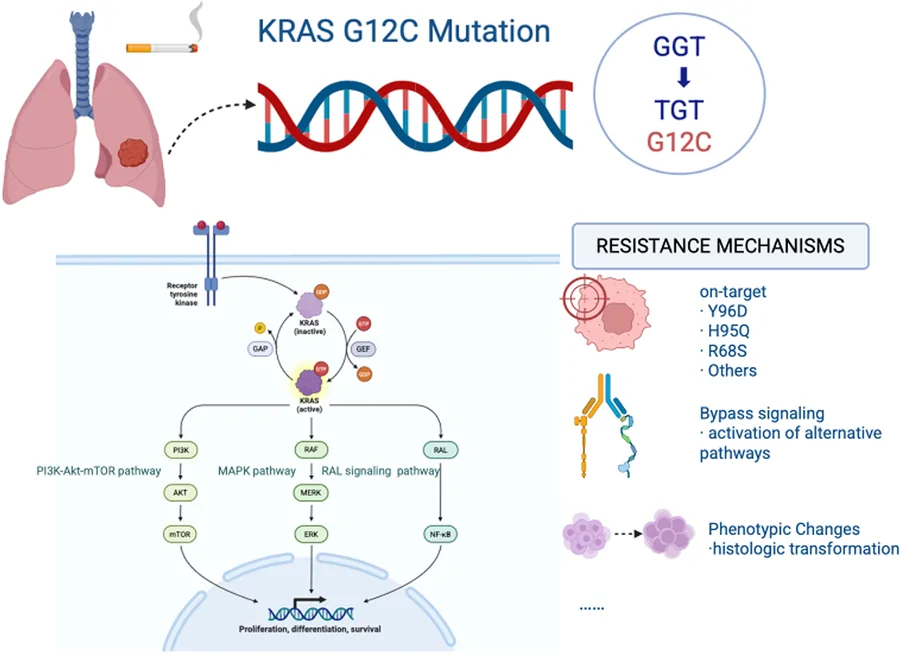

**Abbreviations**
CNS: central nervous system
ctDNA: circulating tumor DNA
DCR: disease control rate
NGS: next‐generation sequencing
NSCLC: non‐small cell lung cancer
ORR: objective response rate
OS: overall survival
PD‐L1: programmed death‐ligand 1
PFS: progression‐free survival
TMB: tumor mutational burden
TTF‐1: thyroid transcription factor 1

## 1 Introduction

KRAS encodes a small GTPase that cycles between an active GTP‐bound and an inactive GDP‐bound state, governing proliferation, metabolism and survival through RAF‐MEK‐ERK, PI3K‐AKT‐mTOR and RAL signaling [1, 2]. The G12C substitution introduces a cysteine at codon 12 that provides a chemically tractable handle for covalent engagement. Identification of the switch‐II pocket in 2013 enabled the clinical development of allele‐selective KRAS G12C inhibitors [3, 4]. Sotorasib and adagrasib established the clinical activity of direct KRAS G12C inhibition in previously treated non‐small cell lung cancer (NSCLC), with subsequent randomized phase 3 trials providing comparative evidence against docetaxel [5, 6, 7, 8]. In China, fulzerasib, garsorasib, and glecirasib have further expanded later‐line treatment options on the basis of pivotal phase 2 studies [9, 10, 11].

Nevertheless, the availability of active drugs does not by itself define an optimal treatment sequence. Five unresolved issues remain central: biological and microenvironmental heterogeneity limits the predictive value of a single mutation; first‐line evidence remains dominated by early‐phase or single‐arm studies; cross‐trial differences in response and PFS are confounded by baseline risk and trial design; resistance is often polyclonal and cannot be managed reliably by empirical within‐class switching; and rapid regulatory progress in China has not eliminated disparities in testing, reimbursement, hospital access or real‐world evidence. This review therefore sets out to separate established clinical practice from investigational strategy, and to argue for mechanism‐guided longitudinal management.

## 2 Epidemiology, Molecular Biology and Clinical Heterogeneity

### 2.1 Epidemiologic and Clinical Characteristics

KRAS alterations occur in approximately 20%–30% of Western lung adenocarcinomas, with G12C usually the most common subtype, whereas prevalence is lower in Asian populations [12]. The LC‐SCRUM‐Asia study reported a KRAS G12C frequency of approximately 4.0% among Asian patients with NSCLC, with enrichment in men and in smokers [13]. Large Chinese genomic and real‐world cohorts have identified KRAS mutations in approximately 9.8%–12.1% of NSCLC cases, with G12C accounting for nearly 30% of KRAS‐mutant tumors [14, 15, 16]. Differences among cohorts may reflect histological composition, stage, tobacco exposure, assay design and enrollment period rather than ancestry alone.

KRAS G12C‐mutant tumors often show relatively high tumor mutational burden (TMB) and PD‐L1 expression, but neither feature guarantees sensitivity to immunotherapy. Advanced KRAS G12C‐mutant disease has also been associated with a relatively high burden of brain metastases, although trials differ in their eligibility criteria for untreated or active central nervous system (CNS) disease. Adagrasib has prospective intracranial activity data, whereas evidence for several other agents is less mature [6, 17, 18]. Baseline assessment should therefore include smoking history, histology, PD‐L1 expression, disease burden and brain MRI when clinically indicated.

### 2.2 Molecular Biology, Co‐Alterations and Tumor Microenvironment

The KRAS G12C mutation impairs GTPase‐activating protein (GAP)‐mediated GTP hydrolysis, so that the protein persists preferentially in its activated state. Classical covalent inhibitors preferentially bind GDP‐bound KRAS G12C and lock the protein in an OFF conformation [3, 19, 20, 21, 22]. Increased upstream RTK‐SHP2‐SOS1 signaling, a higher proportion of GTP‐bound KRAS, or downstream and bypass reactivation can therefore attenuate the activity of monotherapy. The core signaling events and resistance mechanisms are summarized in Figure 1.

**Figure 1:**
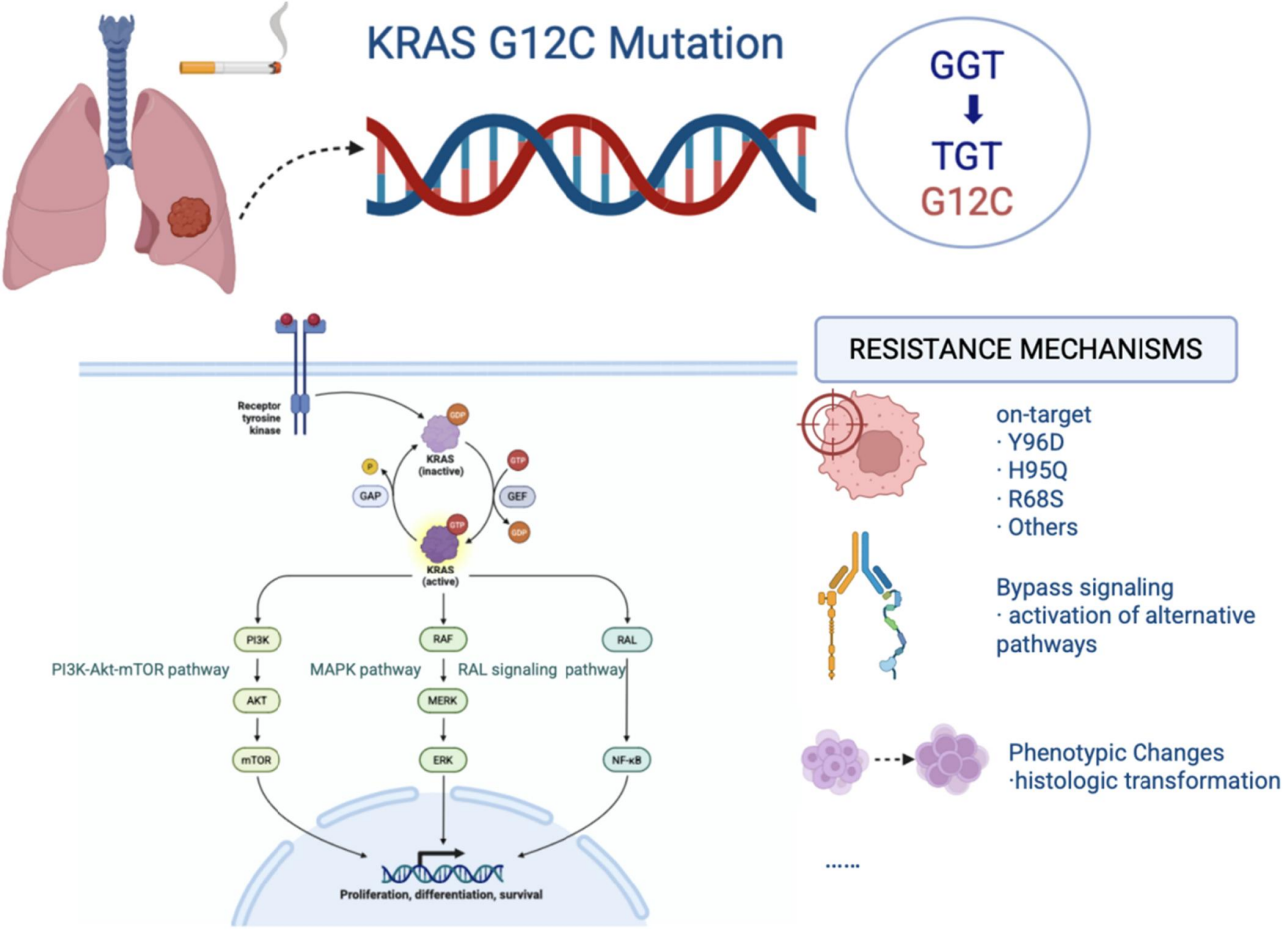
Molecular signaling and major resistance patterns in KRAS G12C‐mutant NSCLC. KRAS G12C cycles between GDP‐bound inactive and GTP‐bound active states and signals through MAPK, PI3K‐AKT‐mTOR and RAL pathways. Resistance may arise from on‐target substitutions, bypass signaling or phenotypic transformation. AKT, protein kinase B; ERK, extracellular signal‐regulated kinase; GAP, GTPase‐activating protein; GDP, guanosine diphosphate; GEF, guanine nucleotide exchange factor; GTP, guanosine triphosphate; KRAS, KRAS proto‐oncogene, GTPase; MAPK, mitogen‐activated protein kinase; MEK, mitogen‐activated protein kinase kinase; mTOR, mechanistic target of rapamycin; NF‐κB, nuclear factor kappa B; PI3K, phosphoinositide 3‐kinase; RAF, rapidly accelerated fibrosarcoma kinase; RAL, RAS‐like small GTPase; RTK, receptor tyrosine kinase.

KRAS G12C is often mutually exclusive with dominant drivers such as EGFR, ALK, and ROS1, but it commonly coexists with TP53, STK11, KEAP1, SMARCA4, and CDKN2A/B alterations [12, 23, 24, 25]. TP53 co‐mutation is often associated with an inflammatory microenvironment, STK11 loss with an immune‐cold phenotype, and KEAP1 loss or NRF2 activation with oxidative‐stress adaptation and poor outcomes from both targeted and immune‐based therapy [24, 25, 26, 27, 28, 29]. Because these associations derive largely from retrospective or exploratory analyses and may be prognostic as much as predictive, none should be used in isolation to withhold an approved regimen. Oncogenic KRAS also reshapes myeloid cells, regulatory T cells, cancer‐associated fibroblasts and tumor metabolism, producing a microenvironment in which high mutational burden may coexist with immune suppression [30, 31]. PD‐L1, TMB, STK11/KEAP1/TP53 status, TTF‐1 expression and ctDNA tumor fraction should therefore be read as a composite portrait rather than as interchangeable single thresholds.

## 3 Molecular Testing, Biomarkers and Access in China

### 3.1 Baseline Tissue and Plasma Testing

For advanced non‐squamous NSCLC and for selected squamous tumors, tissue‐based multigene panel next‐generation sequencing (NGS) remains among the most clinically informative testing strategies for identifying KRAS G12C, other actionable drivers and clinically relevant co‐alterations [32, 33, 34, 35]. Plasma ctDNA‐NGS is a useful alternative or complement when tissue is inadequate, biopsy risk is high, or turnaround time is clinically unacceptable. A negative plasma result may reflect low tumor shedding or low tumor burden and should be confirmed in tissue when possible [36, 37, 38]. PCR and droplet digital PCR can provide rapid or low‐abundance detection but do not define the full co‐mutation landscape. RNA‐based testing is complementary for fusions, rearrangements, splicing events and expression‐dependent alterations, rather than a primary assay for a KRAS point mutation [39, 40, 41].

### 3.2 Dynamic ctDNA and Composite Biomarkers

Liquid biopsy has outgrown its original role as a substitute for tissue sampling and now serves as an important tool for dynamic response assessment and resistance monitoring. ctDNA testing is minimally invasive, repeatable, and capable of reflecting tumor heterogeneity in real time. Early ctDNA decline or clearance during KRAS G12C inhibition is associated with radiographic response and prolonged PFS and may precede imaging progression [28, 42, 43, 44]. No randomized evidence shows that changing treatment solely because of ctDNA kinetics improves survival. Serial ctDNA is therefore best used for risk assessment, prompting reassessment or supporting clinical‐trial decisions rather than as a replacement for RECIST assessment and clinical judgment. Low TTF‐1 expression, KEAP1/NRF2 abnormalities and a high baseline ctDNA tumor fraction are associated with adverse outcomes [28, 45], while spatial transcriptomics, single‐cell omics, and protein target‐engagement assays remain investigational [46, 47, 48].

To improve clinical operability, this review proposes an implementable biomarker framework, shown in Figure 2. Standard first‐line choices should be selected according to PD‐L1 expression, histology, performance status, disease tempo, and contraindications to immunotherapy. STK11/KEAP1/TP53 alterations, TTF‐1 expression, TMB, baseline ctDNA fraction, and brain metastases can refine prognosis and clinical‐trial prioritization, but should not independently determine a specific standard regimen. Selected patients with PD‐L1 ≥ 50%, high TTF‐1 expression, no KEAP1/NRF2 high‐risk signal, and low disease burden may be considered for immune‐checkpoint inhibitor monotherapy within guideline recommendations. Chemoimmunotherapy is generally preferred when PD‐L1 expression is below 50%, rapid tumor reduction is required, or disease burden is high. Adverse molecular features may strengthen the rationale for clinical‐trial enrollment but do not, by themselves, mandate a particular standard regimen. Except for PD‐L1, these biomarkers have not been prospectively validated to select a specific standard drug and should be used for risk stratification and trial enrichment rather than to override guidelines. At disease progression, the framework further matches the pattern of progression to tissue and plasma retesting.

**Figure 2:**
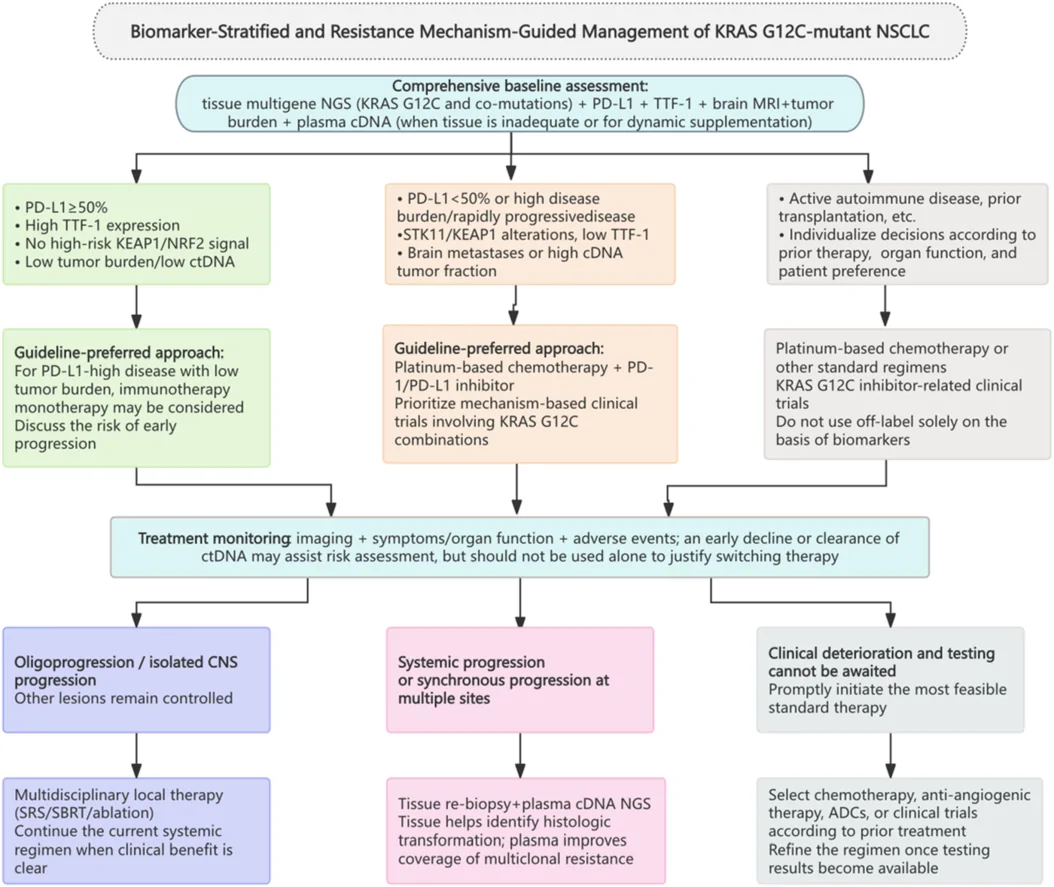
Biomarker‐stratified and resistance mechanism‐guided management of KRAS G12C‐mutant NSCLC. The framework supports integrated risk stratification and clinical trial selection. PD‐L1 is the only mature biomarker that directly informs standard first‐line treatment; TMB, STK11, KEAP1/NRF2, TTF‐1 expression, brain metastases and ctDNA fraction are prognostic or trial‐enrichment variables and should not independently determine a standard regimen. ADC, antibody–drug conjugate; CNS, central nervous system; ctDNA, circulating tumor DNA; KEAP1, Kelch‐like ECH‐associated protein 1; KRAS, KRAS proto‐oncogene, GTPase; MRI, magnetic resonance imaging; NGS, next‐generation sequencing; NRF2, nuclear factor erythroid 2‐related factor 2; NSCLC, non‐smallcell lung carcinoma; PD‐1, programmed cell death protein 1; PD‐L1, programmed death‐ligand 1; SBRT, stereotactic body radiotherapy; SRS, stereotactic radiosurgery; STK11, serine/threonine kinase 11; TTF‐1, thyroid transcription factor 1.

### 3.3 Testing Access, Quality Control and Reimbursement in China

The 2024 Chinese guideline for molecular pathological testing in NSCLC prioritizes tumor tissue, supports cytology or liquid biopsy when tissue is unavailable, and permits multigene RT‐PCR or single‐gene testing where resources are constrained. It emphasizes standardized sampling, parallel testing, quality control and integrated interpretation of tissue and plasma results [38]. Multigene NGS is established in major tertiary cancer centers, but specimen referral, turnaround time, tissue management and out‐of‐pocket cost remain barriers in less‐resourced settings [49]. National reimbursement policy has promoted hospital availability and dual‐channel access for negotiated medicines, although actual patient cost‐sharing is determined locally [50, 51]. Table 1.

**Table 1:** KRAS G12C testing methods, evidentiary role and clinical accessibility in China.

Method	Sample	Main advantages	Main limitations	Evidence/clinical role	Access and payment in China	References
ARMS‐PCR/qPCR	Tissue or plasma	Rapid and relatively inexpensive	Restricted to predefined variants; no co‐mutation profile	Rapid confirmation of known G12C; not a substitute for comprehensive profiling	Widely available; reimbursement and reagent registration vary by institution	[33, 35, 36, 38]
ddPCR/BEAMing	Tissue or plasma	Sensitive quantification of low‐abundance variants	Limited multiplexing; thresholds require validation	Adjunct for dynamic studies or low‐level variants	Specialized laboratories; no validated threshold for treatment switching	[34, 35, 36]
Tissue DNA‐NGS	FFPE or fresh tissue	Simultaneous detection of drivers and co‐mutations	Tissue quantity, quality control, turnaround time and cost	Preferred baseline assay when feasible	Common in tertiary centers; outsourcing and payment vary regionally	[33, 35, 36, 37, 38]
Plasma ctDNA‐NGS	Peripheral blood	Minimally invasive, repeatable and capable of capturing multiclonality	False‐negative results in low‐shedding disease; no histological information	Alternative or complement when tissue is inadequate; progression profiling	Increasing access; negative results require tissue confirmation when feasible	[35, 36, 37, 38, 49]
RNA‐NGS/RT‐PCR	Tissue RNA	Detects fusions, exon‐skipping events and transcript‐level alterations	RNA degradation; not preferred for a KRAS point mutation	Complementary testing for complex molecular profiling	Dependent on sample quality and laboratory capacity	[39, 40, 41]
Multi‐omics/protein engagement assays	Tissue or research specimens	Functional and microenvironmental characterization	High cost, limited standardization and no established thresholds	Exploratory/translational research	Mainly restricted to research platforms; not a routine treatment gate	[25, 46, 47, 48]

Abbreviations: ARMS, amplification‐refractory mutation system; BEAMing, beads, emulsion, amplification and magnetics; ctDNA, circulating tumor DNA; ddPCR, droplet digital PCR; FFPE, formalin‐fixed paraffin‐embedded; NGS, next‐generation sequencing; qPCR, quantitative PCR; RT‐PCR, reverse‐transcription PCR.

## 4 First‐Line Treatment: Current Standards and Investigational Combinations

### 4.1 Conventional Standard Treatment

No randomized phase III trial has shown that first‐line KRAS G12C inhibitor monotherapy or a KRAS G12C inhibitor‐based combination is superior to established treatment for advanced NSCLC without another actionable driver alteration. To date, ASCO and Chinese guidelines generally continue to recommend monotherapy for selected patients with high PD‐L1 expression or platinum‐based chemotherapy plus a PD‐1/PD‐L1 inhibitor according to PD‐L1 expression, performance status, histology, disease burden and immunotherapy contraindications [52, 53, 54, 55]. Chemoimmunotherapy is generally more practical when rapid tumor reduction is required, PD‐L1 expression is low or disease burden is high. Immunotherapy alone may be discussed for selected patients with high PD‐L1 expression who wish to avoid chemotherapy toxicity, after addressing the risk of early progression. Conventional chemotherapy and immunotherapy nevertheless leave the first‐line needs of patients with KRAS G12C‐mutant NSCLC incompletely met, which has driven the rapid development of earlier‐line KRAS G12C inhibitor strategies and combination approaches.

### 4.2 Investigational First‐Line Strategies

First‐line KRAS G12C combinations often yield higher ORR than later‐line monotherapy, but their clinical value is limited by small cohorts, single‐arm designs, selection of evaluable patients, short follow‐up and the absence of a common comparator. These regimens remain investigational pending positive randomized phase III evidence and regulatory approval.

#### 4.2.1 KRAS G12C Inhibitor Combined With Immunotherapy

The combination of KRAS G12C inhibitors with ICIs rests on a clear biological rationale: attenuation of oncogenic signaling, enhanced antigen presentation and improved T‐cell infiltration [4, 56, 57]. Early clinical studies suggest that KRAS G12C inhibitors combined with PD‐1/PD‐L1 inhibitors have antitumor activity, but they have also revealed notable safety concerns. CodeBreaK 100/101 showed frequent transaminase elevation when sotorasib was administered concurrently or sequentially with PD‐1/PD‐L1 inhibitors, indicating that previous immune exposure, sequencing and drug‐specific factors all influence hepatotoxicity [58]. Dose, schedule and the interval from prior immunotherapy therefore require further evaluation to define a safe therapeutic window.

A conference‐reported analysis of the single‐arm phase II KRYSTAL‐7 study showed that first‐line adagrasib plus pembrolizumab for metastatic KRAS G12C‐mutated NSCLC achieved an ORR of approximately 44% and median PFS of approximately 11 months in the overall population, with a stronger response signal in the PD‐L1 ≥ 50% subgroup [59]. In addition to adagrasib, olomorasib combined with pembrolizumab has also shown preliminary activity [60, 61, 62, 63].

#### 4.2.2 Chemotherapy, Anti‐Angiogenic Therapy and Multi‐Drug Intensification

Combining targeted therapy with platinum‐based chemotherapy may couple rapid cytoreduction with sustained driver suppression. Representative studies include CodeBreaK 101 and SCARLET. CodeBreaK 101 showed that sotorasib plus carboplatin/pemetrexed achieved an ORR of 65% and median PFS of 10.8 months, at the cost of substantial hematological toxicity [64, 65]. The SCARLET (WJOG14821L) study reported an ORR of 88.9%, but median PFS of only 6.6 months, illustrating that a high response rate may not translate into durable control [66]. Randomized evaluation is being pursued in CodeBreaK 202, which compares first‐line sotorasib with pembrolizumab plus platinum‐doublet chemotherapy in patients with PD‐L1‐negative, locally advanced or metastatic KRAS G12C‐mutant NSCLC [67].

Preclinical and translational data provide a rationale for combining targeted therapy with antiangiogenic treatment [68, 69]. The SHERLOCK study evaluated sotorasib combined with carboplatin/pemetrexed and a bevacizumab biosimilar as first‐line treatment and showed a confirmed ORR of approximately 64% and median PFS of approximately 9.0 months, with response signals observed across different PD‐L1 subgroups, together with ctDNA clearance signals [70]. In an integrated analysis of LOXO‐RAS‐20001 and the safety lead‐in/dose‐optimization portion of SUNRAY‐01, first‐line olomorasib plus pembrolizumab, pemetrexed, and platinum chemotherapy showed encouraging antitumor activity in advanced KRAS G12C‐mutated NSCLC, with responses observed across PD‐L1 subgroups [61, 62, 63]. The potential for cumulative myelosuppression, hepatotoxicity, immune‐related adverse events and anti‐angiogenic toxicity must be weighed against early efficacy signals.

#### 4.2.3 Dual‐Targeted Combination Strategies: EGFR and FAK Pathways

RTK feedback and the adhesion‐FAK‐YAP axis provide a rationale for dual targeting. In the single‐arm phase Ib/II KROCUS study, first‐line fulzerasib plus cetuximab achieved a confirmed ORR of 68.9% and median PFS of 12.5 months [71]. These results suggest that EGFR‐mediated feedback is clinically actionable, although skin, gastrointestinal and infusion‐related toxicities, together with the absence of a standard comparator, limit the strength of the conclusions. A Chinese phase Ib/II expansion cohort of garsorasib plus the FAK inhibitor ifebemtinib also reported an ORR of 82% and median PFS of 22.3 months in 33 patients [72, 73]. These studies provide biological validation but cannot establish a new standard, given small sample sizes, single‐arm designs, limited follow‐up and the added burden of EGFR‐ or FAK‐related toxicity.

Although combination strategies have shown preliminary efficacy signals, the positioning of KRAS G12C inhibitors in first‐line therapy remains controversial. The optimal target populations, sequential strategies, and comparative value of these combination regimens versus standard monotherapy still require validation in prospective randomized studies, and the challenges of toxicity management persist. Table 2 provides a systematic comparison of first‐line regimens for KRAS G12C‐mutated NSCLC. Divarasib (GDC‐6036) is an oral, highly selective KRAS G12C inhibitor that has likewise shown activity as monotherapy in untreated subgroups, but the numbers are small and should not be compared directly with combination cohorts [74, 75].

**Table 2:** Systematic comparison of first‐line strategies for KRAS G12C‐mutant NSCLC.

Strategy/representative study	Phase and design	Main efficacy signal	Principal toxicity or limitation	Evidence level	Current role
Standard ICI monotherapy or chemoimmunotherapy [52, 53, 54, 55]	Randomized phase III evidence in non‐oncogene‐addicted NSCLC	Established survival benefit in driver‐negative NSCLC	Evidence is extrapolated to the KRAS subtype; immune and chemotherapy toxicities	I	Routine first‐line standard
Adagrasib + pembrolizumab (KRYSTAL‐7) [59]	Phase II, single arm	ORR about 44%; median PFS about 11 months	No randomized comparator; frequent grade 3/4 treatment‐related events, including hepatotoxicity	II	Investigational; randomized validation pending
Sotorasib + carboplatin/pemetrexed (CodeBreaK 101) [64, 65]	Phase Ib dose expansion	First‐line ORR about 65%; median PFS about 10.8 months	Small cohort; myelosuppression	III	Investigational
Sotorasib + carboplatin/pemetrexed (SCARLET) [66]	Phase II, single arm	ORR 88.9%; median PFS 6.6 months	Discordance between high ORR and PFS; small cohort and hematological toxicity	II	Investigational
Sotorasib + chemotherapy + bevacizumab (SHERLOCK) [70]	Phase II, single arm, conference data	ORR about 64%; median PFS about 9 months	Cumulative four‐drug toxicity; no comparator	III	Investigational
Olomorasib + pembrolizumab ± chemotherapy [62, 63]	Phase I/II safety lead‐in and dose optimization	Responses across PD‐L1 subgroups	Mature PFS, OS and comparative data unavailable	III	Phase III evaluation ongoing
Fulzerasib + cetuximab (KROCUS) [71]	Phase Ib/II multicentre single arm	ORR 68.9%; median PFS 12.5 months	EGFR‐related toxicity; no standard comparator	II	Investigational dual targeting
Garsorasib + ifebemtinib [73]	Phase Ib/II single‐arm expansion in China	High ORR and encouraging PFS signal	Small cohort	II	Investigational dual targeting
Divarasib monotherapy/combination [74, 75]	Phase I and subsequent pivotal studies	Untreated subgroup: ORR about 55.6%; median PFS about 15.3 months	Small subgroup; cross‐trial comparison invalid	III	Pivotal randomized studies ongoing

*Note:* ORR and PFS values originate from different studies and should not be used for non‐randomized ranking. Evidence levels: I, randomized phase III trials, guidelines, or regulatory standards; II, prospective phase II studies, including single‐arm studies; III, phase I/Ib studies, dose‐expansion cohorts, or conference abstracts; IV, retrospective, real‐world, translational, or preclinical evidence.

Abbreviations: ICI, immune‐checkpoint inhibitor; ORR, objective response rate; PFS, progression‐free survival; NSCLC, non‐small cell lung cancer.

## 5 Later‐Line Targeted Therapy and Implementation in China

### 5.1 International Randomized Evidence

CodeBreaK 100 established later‐line activity for sotorasib. In the randomized phase III CodeBreaK 200 trial, sotorasib improved PFS and ORR compared with docetaxel, but did not significantly improve OS. Crossover and the statistical design of the trial complicate interpretation [5, 7]. It therefore establishes a better‐tolerated targeted option with a PFS benefit, rather than a proven extension of survival. KRYSTAL‐1 reported an ORR of 42.9% and median PFS of 6.5 months for adagrasib and demonstrated prospective intracranial activity in patients with untreated CNS metastases [6, 18]. The randomized phase III KRYSTAL‐12 trial confirmed superior PFS and ORR versus docetaxel [8]. Sotorasib and adagrasib received FDA approval in 2021 and 2022, respectively, for locally advanced or metastatic KRAS G12C‐mutant NSCLC after at least one previous systemic therapy [76].

### 5.2 Approved Agents and Real‐World Implementation in China

Chinese KRAS G12C drug development has progressed from confirmatory follow‐up to domestic competition and combination development. Fulzerasib, garsorasib, glecirasib, and sosimerasib received conditional approval in August 2024, November 2024, May 2025, and February 2026, respectively, for adults with advanced KRAS G12C‐mutant NSCLC after at least one systemic regimen [77, 78, 79, 80]. Peer‐reviewed pivotal single‐arm phase II data are available for the first three agents [9, 10, 11]. Conditional approval accelerates access but does not discharge the obligation to complete confirmatory trials, long‐term safety surveillance and post‐marketing reassessment.

The 2025 National Reimbursement Drug List took effect on January 1, 2026 and includes fulzerasib, glecirasib and garsorasib for previously treated advanced KRAS G12C‐mutant NSCLC [50, 51]. Sosimerasib has entered the application and preliminary review process for the 2026 adjustment, but the final decision was not available at the literature cut‐off [81]. Reimbursement can reduce drug cost, whereas hospital formulary entry, dual‐channel access and patient co‐payment still vary by region. National real‐world databases are needed to evaluate outcomes in patients with brain metastases, poor performance status, older age, organ dysfunction or complex co‐alterations; hepatotoxicity after immunotherapy; treatment delay and adherence after reimbursement; and whether sequential use of different inhibitors provides clinical benefit. Table 3 lists the key evidence, regulatory status, and accessibility in China of currently approved KRAS G12C inhibitors domestically and internationally.

**Table 3:** Key evidence, regulatory status, and accessibility of approved KRAS G12C inhibitors.

Agent	Key study/design	Main NSCLC signal	Evidence level	Regulatory status	Access and reimbursement in China	Reference
Sotorasib	CodeBreaK 100 (phase II single arm); CodeBreaK 200 (phase III randomized)	ORR 37.1%, median PFS 6.8 months; randomized PFS benefit over docetaxel without proven OS benefit	I	US accelerated approval for previously treated disease	Not among the three domestic agents listed in the 2025 NRDL;	[5, 7, 50, 51, 76]
Adagrasib	KRYSTAL‐1 (phase I/II); KRYSTAL‐12 (phase III randomized)	ORR 42.9%, median PFS 6.5 months; randomized PFS benefit over docetaxel; prospective intracranial activity	I	US accelerated approval for previously treated disease	Not among the three domestic agents listed in the 2025 NRDL;	[6, 8, 18, 50, 51, 76]
Fulzerasib (IBI351)	Registrational multicentre phase II single‐arm study	ORR 49.1%; DCR 90.5%; median PFS 9.7 months	II	NMPA conditional approval (August 2024)	Included in the 2025 NRDL	[9, 50, 51, 77]
Garsorasib (D‐1553)	Multicentre phase II single‐arm study in China	ORR 52.0%; DCR 88.6%; median PFS about 9.1 months	II	NMPA conditional approval (November 2024)	Included in the 2025 NRDL	[10, 50, 51, 78]
Glecirasib (JAB‐21822)	Multicentre phase IIb single‐arm study	ORR about 48%–50%; DCR 86.3%; median PFS 8.2 months	II	NMPA conditional approval (May 2025)	Included in the 2025 NRDL	[11, 50, 51, 79]
Sosimerasib	Registrational study/regulatory data	Public data support later‐line activity; long‐term and confirmatory evidence remain limited	II–III	NMPA conditional approval (February 2026)	Not in the 2025 NRDL; entered preliminary review for the 2026 adjustment	[80, 81]

*Note:* Single‐arm studies cannot be compared directly. Indications, dosing and reimbursement should follow the latest label and national or local reimbursement policy.

Abbreviations: DCR, disease control rate; NMPA, National Medical Products Administration; NRDL, National Reimbursement Drug List; NSCLC, non‐small cell lung cancer; ORR, objective response rate; OS, overall survival; PFS, progression‐free survival.

### 5.3 Why Efficacy Estimates Differ Across KRAS G12C Inhibitors

ORR and median PFS differ across studies, but no head‐to‐head randomized trial has shown that one KRAS G12C inhibitor is superior to another. Even for the same drug, ORR may be lower in a randomized intention‐to‐treat population than in a single‐arm registration cohort: sotorasib, for example, achieved 37.1% in CodeBreaK 100 but approximately 28.1% in CodeBreaK 200, whereas adagrasib achieved 42.9% in KRYSTAL‐1 and 32% in KRYSTAL‐12 [5, 6, 7, 9]. Study phase, analysis population, and trial design therefore contribute substantially to numerical differences.

Pharmacologic properties may also contribute. Relevant differences include switch‐II pocket contacts, biochemical potency and selectivity, target occupancy, half‐life, dose schedule, CNS exposure, metabolism, and drug‐drug interactions. Adagrasib has a half‐life of approximately 23 h and prospective intracranial data; divarasib was designed as a potent and selective OFF‐state inhibitor and has a mean half‐life of approximately 17.6 h with once‐daily dosing [6, 18, 74, 75]. Secondary substitutions at H95, R68 and Y96 may differentially affect individual inhibitors [82, 83]. These observations offer plausible explanations for the differences observed; they are not evidence of comparative clinical superiority.

Current selection should therefore be based on local approval and randomized evidence, prospective CNS data, recent immunotherapy exposure and hepatotoxicity risk, gastrointestinal, hepatic and cardiac comorbidity, drug interactions, dosing frequency, adherence, reimbursement and sustained supply. Domestically developed agents should be selected according to the approved prescribing information, access and individual safety considerations, rather than ranked by single‐arm ORR.

## 6 Resistance and Mechanism‐Guided Management

### 6.1 Primary and Acquired Resistance

Although KRAS G12C inhibitors have substantially advanced precision therapy for KRAS‐mutant NSCLC, the duration of clinical benefit remains decisively limited by resistance, which is conventionally classified as primary or acquired. Primary resistance is commonly described as progression at the first assessment or PFS shorter than approximately 3 months, although this cutoff is neither uniform nor absolute. Progression after an initial period of control is categorized as acquired resistance. Primary resistance may arise from a high KRAS‐GTP fraction, RTK‐SHP2‐SOS1 dependence, KEAP1/NRF2 abnormalities, epithelial‐mesenchymal transition (EMT), stem‐like states or incomplete dependence on KRAS G12C [28, 29, 84, 85, 86, 87, 88, 89, 90, 91]. Acquired resistance is usually a dynamic, polyclonal process and may differ across metastatic sites [82, 83, 92, 93, 94].

### 6.2 On‐Target, Bypass and Phenotypic Resistance

On‐target resistance involves secondary KRAS alterations that impair inhibitor binding or increase KRAS signaling output, such as R68S, H95D/Q/R, Y96C/D/S, G13D, and Q61H, as well as KRAS G12C amplification [82, 83, 92]. Among these, Y96D/Y96S may disrupt switch‐II pocket engagement and confer cross‐resistance, whereas other secondary mutations may retain differential sensitivity to distinct KRAS G12C inhibitors. The available evidence is largely preclinical or drawn from case reports and does not support empirical within‐class switching.

Off‐target resistance is mainly driven by bypass and downstream pathway activation, including EGFR/MET‐mediated RTK signaling, SOS1/SHP2 feedback, NRAS, BRAF, MAP2K1 or RAF1 alterations, PI3K‐AKT‐mTOR signaling and adaptive AXL, YAP/TAZ, FAK or MRAS‐SHOC2‐PP1C activity [87, 93, 95, 96, 97, 98, 99, 100, 101, 102, 103, 104, 105]. Histological transformation, EMT and adeno‐to‐squamous transition require re‐biopsy and cannot be reliably identified by plasma testing alone [90, 91, 106].

### 6.3 Progression Pattern, Re‐Biopsy and Subsequent Intervention

Management after resistance should begin with the pattern of progression rather than automatic substitution of another KRAS inhibitor. Selected patients with oligoprogression, particularly when extracranial disease remains controlled, may receive stereotactic radiotherapy or ablation while continuing the current systemic regimen. This approach is extrapolated from broader NSCLC data rather than proven specifically for KRAS G12C inhibition [107]. Systemic progression should prompt tissue re‐biopsy and/or plasma ctDNA‐NGS. Tissue identifies transformation, microenvironmental features and protein expression, while plasma captures multiclonal alterations across lesions.

Mechanism‐matched treatment should preferably be delivered in clinical trials. MET amplification or EGFR/other RTK activation provides a rationale for matched RTK combinations; SHP2/SOS1 feedback, downstream MAPK reactivation and PI3K/AXL/YAP/FAK bypass signaling support corresponding investigational combinations [95, 98, 99, 100, 101, 102, 103, 104, 105]. Y96 or related pocket alterations are better matched to RAS(ON), ON/OFF or other next‐generation inhibitor trials than to blind switching among OFF‐state inhibitors. When no actionable mechanism is identified, treatment should reflect prior exposure and may include docetaxel with or without ramucirumab, another chemotherapy regimen, an antibody‐drug conjugate or a clinical trial [108, 109]. Re‐biopsy findings must be interpreted together with clonal abundance, progression pattern and previous therapy, and all combinations require assessment of overlapping toxicity. Post‐resistance care should accordingly shift from empirical drug switching toward dynamic management grounded in molecular stratification, mechanistic attribution and individualized combination therapy. Figure 3.

**Figure 3:**
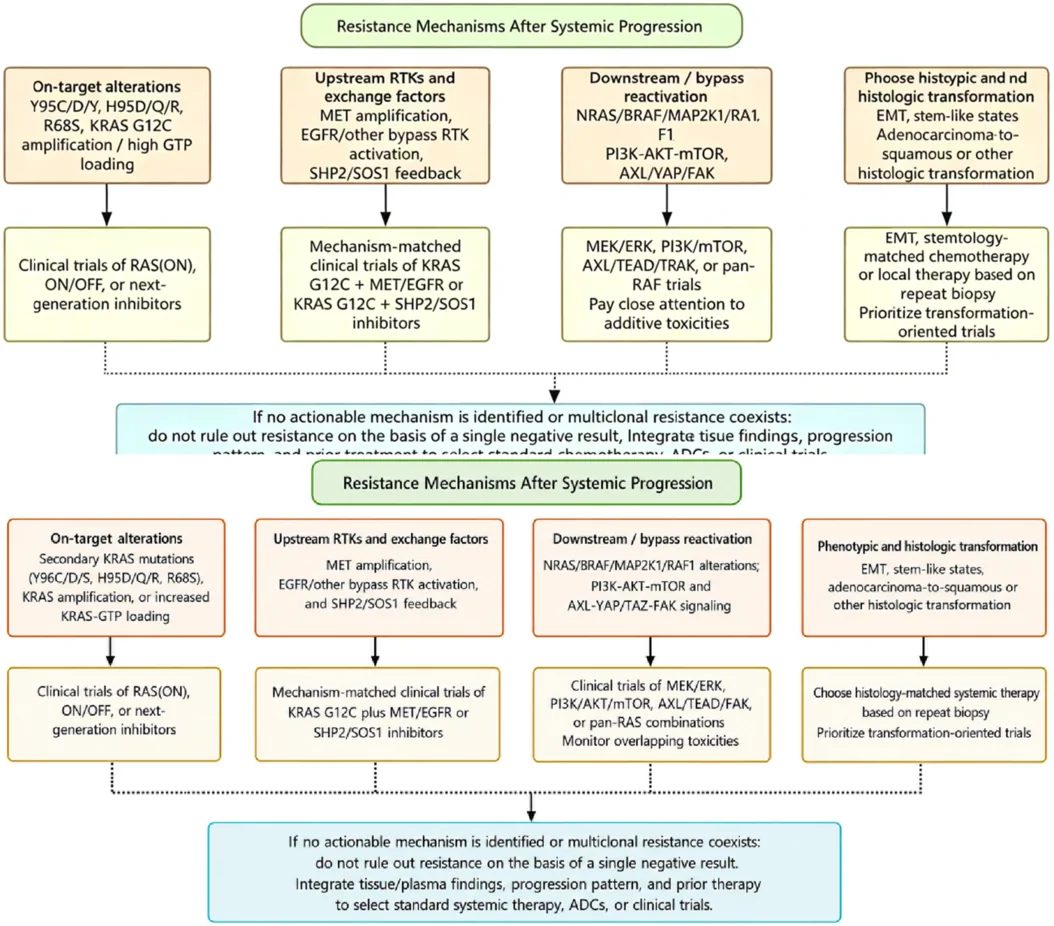
Mechanism‐directed management of KRAS G12C‐mutant NSCLC drug resistance. ADC, antibody–drug conjugate; AKT, protein kinase B; AXL, AXL receptor tyrosine kinase; BRAF, B‐Raf proto‐oncogene, serine/threonine kinase; EGFR, epidermal growth factor receptor; EMT, epithelial–mesenchymal transition; ERK, extracellular signal‐regulated kinase; FAK, focal adhesion kinase; GTP, guanosine triphosphate; KRAS, KRAS proto‐oncogene, GTPase; MAP2K1, mitogen‐activated protein kinase kinase 1; MEK, mitogen‐activated protein kinase kinase; MET, mesenchymal–epithelial transition factor; mTOR, mechanistic target of rapamycin; NRAS, neuroblastoma RAS viral oncogene homolog; PI3K, phosphoinositide 3‐kinase; RAF1, RAF proto‐oncogene serine/threonine‐protein kinase; RAS, rat sarcoma; RTK, receptor tyrosine kinase; SHP2, Src homology 2 domain‐containing protein tyrosine phosphatase 2; SOS1, son of sevenless 1; TAZ, transcriptional coactivator with PDZ‐binding motif; TEAD, TEA domain transcription factor; YAP, Yes‐associated protein.

## 7 Novel Drug Development and Key Problems Requiring Validation. Abbreviations

Future development should move beyond structural novelty for its own sake and address the limitations that are clinically actionable: adequate inhibition of active KRAS‐GTP, coverage of secondary KRAS mutations or KRAS amplification, improving CNS exposure and preserving a sufficient safety window in combination regimens, biomarker‐guided patient selection, expanding target coverage beyond KRAS G12C to G12D, G12V and broader pan‐KRAS/pan‐RAS strategies [18, 60, 92, 94, 110].

### 7.1 RAS(ON) and Dual‐State Inhibitors

BBO‐8520 was designed to engage both ON‐ and OFF‐state KRAS G12C [111]. Elironrasib (RMC‐6291) forms a cyclophilin A‐dependent tri‐complex that targets active KRAS G12C [112, 113]. These approaches may reduce dependence on the GDP‐bound fraction and retain activity after some OFF‐state resistance, but clinical efficacy, resistance spectra, CNS activity and wild‐type RAS toxicity require definition.

### 7.2 Pan‐KRAS/Pan‐RAS and Non‐G12C Inhibitors, and Protein Degradation

MRTX1133 is a representative selective KRAS G12D inhibitor supported predominantly by preclinical evidence, whereas HRS‐4642 has shown early phase I clinical activity [114, 115]. BI‐2865 and BI‐2493 are preclinical pan‐KRAS inhibitors, while daraxonrasib/RMC‐6236 is a multi‐selective RAS(ON) inhibitor with emerging clinical data in RAS‐mutant cancers [116, 117, 118, 119, 120]. Targeted protein degradation (TPD) has evolved from the VHL‐recruiting PROTAC LC‐2 to small‐molecule pan‐KRAS degraders such as ACBI3, providing a complementary approach that eliminates the KRAS protein rather than merely inhibiting its activity [121, 122, 123]. Nevertheless, substantial challenges remain, including pharmacokinetics, tumor selectivity, the dependence of normal tissues on RAS signaling, and long‐term safety. SHP2 or SOS1 combinations seek to suppress upstream feedback and deepen KRAS pathway inhibition, but therapeutic windows and patient selection remain unsettled [98, 99, 100, 124]. Table 4.

**Table 4:** Representative emerging KRAS/RAS strategies and key questions.

Class/representative agent	Target state	Development stage/evidence	Potential value	Key unresolved question	References
Divarasib, olomorasib	KRAS G12C (OFF)	Early clinical to phase III	Optimized potency/selectivity and combinations	Can they outperform standard therapy, with durable CNS control and acceptable long‐term toxicity	[60, 61, 62, 63, 74, 75]
Elironrasib (RMC‐6291)	RAS G12C(ON)	Early clinical	Targets the active state and may overcome selected OFF‐state resistance	Clinical resistance spectrum and combination safety window	[112, 113]
BBO‐8520	KRAS G12C(ON/OFF)	Early clinical/translational	Reduces conformational dependence	Does dual‐state inhibition produce deeper and more durable suppression	[111]
SHP2/SOS1 combinations	Upstream feedback nodes	Early clinical/preclinical	Blocks RTK feedback and increases the GDP‐bound fraction	Therapeutic window; patient selection	[98, 99, 100]
MRTX1133, HRS‐4642	KRAS G12D	Preclinical to early clinical	Extends targeting beyond G12C	Pharmacokinetics, resistance and tissue‐specific activity	[114, 115]
BI‐2865/BI‐2493, daraxonrasib	Pan‐KRAS/pan‐RAS or RAS(ON)	Preclinical to clinical	Covers multiple alleles and pathway reactivation	Therapeutic window for wild‐type RAS inhibition	[116, 117, 118, 119, 120]
LC‐2, ACBI3 and related degraders	KRAS G12C or pan‐KRAS protein	Preclinical	Protein elimination may overcome occupancy limitations	Cell permeability, E3 ligase dependence and human safety	[121, 122, 123]

Abbreviations: GDP, guanosine diphosphate; RAS, rat sarcoma; RTK, receptor tyrosine kinase.

## 8 Conclusions and Perspectives

Once a byword for undruggability, KRAS G12C has become one of the defining successes of precision oncology in advanced NSCLC; the most reliable evidence, however, remains a PFS and ORR benefit in previously treated disease. Randomized studies have not established a consistent OS advantage, and first‐line use and most combination strategies remain investigational. Observed differences among inhibitors reflect the combined effects of binding mode, pharmacodynamics, pharmacokinetics, CNS exposure, tolerability, dose intensity, trial design and baseline patient characteristics; single‐arm ORR should not be used to rank agents.

Clinical management should move from a single‐mutation paradigm toward longitudinal, composite stratification. Baseline tissue NGS, selective use of plasma ctDNA, integration of PD‐L1, TMB, co‐alterations, TTF‐1, brain metastases and ctDNA burden, and re‐biopsy at progression can support a longitudinal framework of molecular stratification, dynamic monitoring and mechanism‐informed intervention. In China, four domestic approvals and reimbursement of three agents have substantially improved access, but testing payment, hospital availability, regional implementation and confirmatory or real‐world evidence will determine their ultimate value. National clinicogenomic databases, randomized first‐line comparisons and paired tissue–plasma resistance studies are priorities for transforming KRAS G12C‐mutant NSCLC from a targetable genotype into sustainable precision care. The field has moved past asking whether KRAS can be inhibited; what remains to be settled is how that inhibition can be made to last, and the answer will depend less on the next molecule than on the rigor with which patients are stratified, monitored and re‐characterized over time.

## Author Contributions

**Haiying Wang:** writing – original draft, writing – review and editing. **Hanxiao Chen:** writing – review and editing, supervision. **Sheng Li:** supervision, funding acquisition, writing – review and editing. **Jun Zhao:** writing – review and editing, conceptualization, funding acquisition, supervision.

## Ethics Statement

The authors have nothing to report.

## Consent

The authors have nothing to report.

## Conflicts of Interest

The authors declare no conflicts of interest.

## Data Availability

Data sharing is not applicable to this article as no datasets were generated or analyzed during the current study.
